# The Role of the Immune System in Huntington's Disease

**DOI:** 10.1155/2013/541259

**Published:** 2013-07-15

**Authors:** Gisa Ellrichmann, Christiane Reick, Carsten Saft, Ralf A. Linker

**Affiliations:** ^1^Department of Neurology, St. Josef-Hospital, Ruhr-University Bochum, 44791 Bochum, Germany; ^2^International Graduate School of Neuroscience, Ruhr-University Bochum, 44791 Bochum, Germany; ^3^Department of Neurology, Friedrich-Alexander-University Erlangen, 91054 Erlangen, Germany

## Abstract

Huntington's disease (HD) is characterized by a progressive course of disease until death 15–20 years after the first symptoms occur and is caused by a mutation with expanded CAG repeats in the huntingtin (htt) protein. Mutant htt (mhtt) in the striatum is assumed to be the main reason for neurodegeneration. Knowledge about pathophysiology has rapidly improved discussing influences of excitotoxicity, mitochondrial damage, free radicals, and inflammatory mechanisms. Both innate and adaptive immune systems may play an important role in HD. Activation of microglia with expression of proinflammatory cytokines, impaired migration of macrophages, and deposition of complement factors in the striatum indicate an activation of the innate immune system. As part of the adaptive immune system, dendritic cells (DCs) prime T-cell responses secreting inflammatory mediators. In HD, DCs may contain mhtt which brings the adaptive immune system into the focus of interest. These data underline an increasing interest in the peripheral immune system for pathomechanisms of HD. It is still unclear if neuroinflammation is a reactive process or if there is an active influence on disease progression. Further understanding the influence of inflammation in HD using mouse models may open various avenues for promising therapeutic approaches aiming at slowing disease progression or forestalling onset of disease.

## 1. Introduction

Huntington's disease (HD) is an autosomal dominantly inherited disorder with a trinucleotide CAG repeat expansion ≥36 in the exon 1 of the HD gene located on chromosome 4 [1]. The unstable CAG repeat is translated into a polyglutamine (polyQ) stretch in the huntingtin (htt) protein, which is ubiquitously expressed, including wide expression in neurons and glial cells [2–7]. The number of CAG repeats negatively correlates with the age of onset of the disease [8, 9].

The mutation leads to involuntary movement disturbances, psychiatric symptoms, and cognitive decline. The degenerative process primarily involves medium spiny striatal neurons and cortical neurons leading to dysfunction and subsequently neuronal loss. 

Since the identification of the HD mutation in 1993, the understanding of the pathophysiology and molecular biology of the disease has significantly improved. Beside others, mechanisms of tissue damage in HD comprise excitotoxicity, mitochondrial damage, free radicals, and possibly also inflammatory mechanisms including microglia activation. New therapeutic strategies aim at slowing disease progression or forestalling the onset of disease. However, it is still unclear if neuroinflammation in HD is only a reactive process or if there is an active influence on disease progression.

Common transgenic murine models of HD are divided into three classes. First, there are fragment models with a human exon 1 N-terminal fragment with about 144 CAG-repeats, for example, the widely used R6/2 model [10]. Second, knock-in mouse models have been generated by introduction of a pathological CAG-repeat into the mouse htt gene [11]. Hdh^Q150/Q150^ mice exemplarily belong to this group [12]. Third, full-length transgenic mouse models express mutant huntingtin (mhtt) on a yeast artificial chromosome (YAC) or bacterial artificial chromosome (BAC). YAC128 mice represent this category [13, 14]. 

The R6/2 and YAC128 mouse strains are well-characterized animal models mimicking many histopathological aspects of HD [10, 15]. In R6/2 mice, motor symptoms start at the age of about 6 weeks. Continuous weight loss leads to death between 11–14 weeks of age. In YAC128 mice with its full-length mhtt spanning about 120 CAG repeats [14, 16], hypoactivity is first seen at the age of 8 months. Additionally, progressive gait abnormalities, ataxia, hind limb clasping, and a progressive decline in the forced motor function occur over time [14, 17].

This review summarizes the current knowledge about the relation between the immune system and HD as well as the putative role of the adaptive and innate immune system in HD.

## 2. Huntington's Disease and the Immune System

In neurodegenerative diseases like Alzheimer's disease (AD), Parkinson's disease (PD), or amyotrophic lateral sclerosis (ALS), there are many studies demonstrating an involvement of neuroinflammation [18–21]. Yet, in HD, much fewer information is available on these processes to date. 

Inflammation both in the CNS or in the periphery is typically initiated by aberration of the normal healthy state due to, for example, pathological injury, trauma, infection, abnormal folding of proteins, or aggregation of other triggers. Neuroinflammation may be mediated by soluble factors including cytokines, prostaglandins, and nitric oxide (NO) finally resulting in neuronal degeneration. A cellular characteristic of neuroinflammation is the presence of microglial cells, a typical marker for immune activation in the CNS [22].

**Figure 1:**
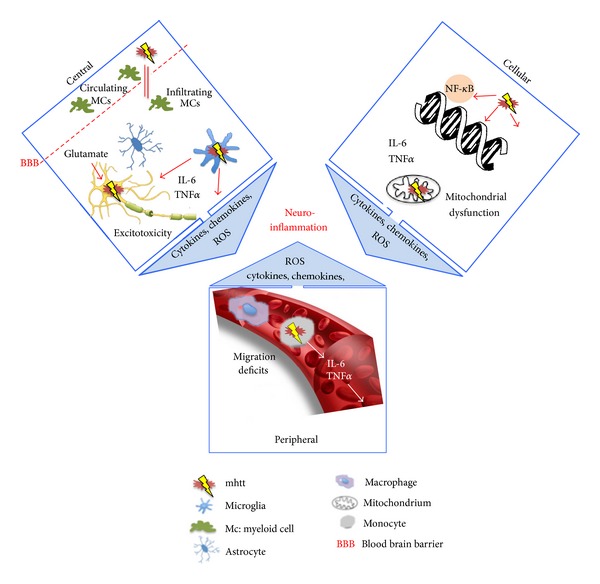
Neuroinflammation in the center of HD pathophysiology. Immune activation, induced by mutant huntingtin (mhtt), is found ubiquitously: in the central nervous system (central), the blood circulation (peripheral), and at molecular level (cellular). In the CNS, mhtt may not only influence migration of cells, for example, myeloid cells, but also induces microglia activation. Cytokines and chemokines (e.g., IL-6, TNF*α*) are secreted, and reactive oxygen species (ROS) are activated. Furthermore, glutamate-induced excitotoxicity, that is, in close interaction with oxidative stress, may contribute to degeneration. Migration deficits are discussed to influence innate immune response in the periphery very early in HD. Once again, cytokines, chemokines, and ROS in concert may trigger neuroinflammation. On a cellular level, mhtt upregulates the NF-kappa B (NF-*κ*B) signaling pathway that triggers IL-6 expression. Finally, mitochondrial dysfunction generated by mhtt seems to be a key player leading to neuroinflammation in HD. The complement system is a connection between the innate and adaptive immune response. There are two ways that activate the complement system: first, inflammation with all its resulting effects targets factors of the complement system. Second, antibody response and T-cell response follow in secretion of complement factors. Various cell types may be affected, among these monocytes, astrocytes, and T and B cells.

A number of studies indicate that activation of the immune system and an altered immune response in HD is evident even in the premanifest stage of the disease [23]. Thus, striatal and cortical neurodegeneration may be triggered by inflammation. Conversely, increased inflammation in HD might be the response to neuronal death induced by mhtt toxicity. Probably, even mhtt itself may trigger inflammation in HD (Figure 1). 

In addition, inflammatory processes may also be caused via an involvement of mitochondrial dysfunction which is a well-known part in the pathophysiology of HD [24–27] (Figure 1). This hypothesis is supported by recent findings in *Candida albicans*, demonstrating a reduced recognition by macrophage receptors and decreased expression of cytokines such as interleukin-(IL-) 6, IL-10, and interferon-(IFN-) *γ* with changes in mitochondrial complex I structure [28]. Moreover, oxidative stress is discussed to induce an assembly of multiprotein inflammatory complexes called “inflammasomes” in normal aging [29], and mitochondrial dynamics influence T-cell function [30]. A link between inflammatory processes and mitochondrial function is also supported by the observation of severe ultrastructural mitochondrial changes in lymphoblasts from patients carrying a homozygous mutation for HD [31]. Glutamate-induced excitotoxicity stands in close relation to mitochondrial damage and also oxidative stress [32, 33]. N-Methyl-D-aspartate (NMDA) receptors are mainly activated by glutamate and are known to be linked to nitric oxide synthases (NOS). This results in increased nitric oxide (NO) levels in toxic amounts [34] leading once again to mitochondrial dysfunction and, in the end, cell death [35]. mhtt may enhance NMDA receptor-mediated toxicity strengthening the vicious circle of neurodegeneration [36] (Figure 1).

Another hypothesis aims at migration deficits of immune cells in HD. An impaired migration may negatively influence cytokines and chemokines resulting in chronically increased levels of proinflammatory cytokines and chemokines in the CNS. In turn, this may lead to microglial activation, neuroinflammation, and finally neurodegeneration in HD [37].

In contrast to inflammatory neurological diseases, for example, multiple sclerosis, there is a marginal influx of peripheral immune cells (lymphocytes, neutrophils) in HD. Based on the fact that T cells are numerically rare in HD pathology [38], major culprits in HD associated with neuroinflammation seem to be microglia, neurons, and macroglia. 

## 3. Role of the Innate Immune System in Huntington's Disease

### 3.1. Microglia

Microglia are primary mediators of neuroinflammation [39, 40] and seem to be key players in the pathogenesis of neurodegenerative diseases [20].

**Figure 2:**
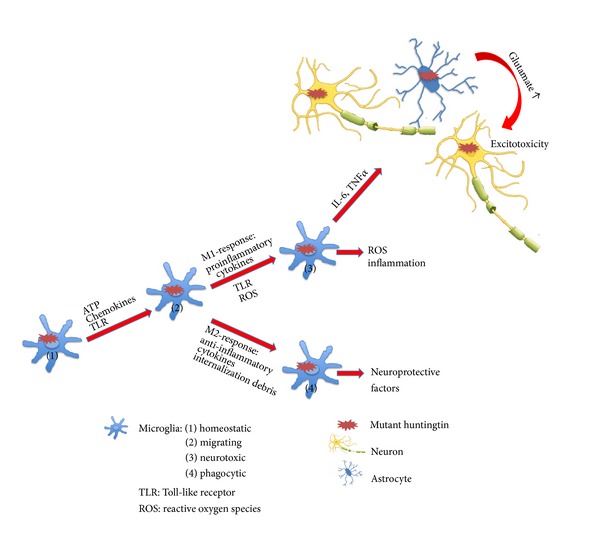
Neuronal glia interactions in Huntington's disease. Microglia/macrophages are essential for host defense and can trigger M1 or M2 responses. The dichotomous M1/M2 concept implies that M1 cells induce Th1 cells that produce proinflammatory cytokines like IFN-*γ*, while M2 cells induce Th2 cells that are associated with IL-4, IL-5, and IL-10 supporting anti-inflammatory effects or antibody production. Aggregates of mutant huntingtin (mhtt) are found in neurons, astrocytes, and microglia. With a decreased protective function due to fewer glutamate transporters, mhtt-containing astrocytes may contribute to excitotoxicity. Consequently, there is glutamate excitotoxicity and glutamate-induced apoptosis causing neurodegeneration. At the same time, mhtt enhances microglia function and leads to microglia activation. Migrating microglia secrete proinflammatory cytokines (e.g., TNF*α*) and nitric oxide thus contributing to neurotoxicity. Yet, simultaneously, phagocytic microglia may also produce neuroprotective factors.

In HD, the main neuropathological changes take place in the nuclei of the basal ganglia and are characterized by degeneration and neuronal loss. Consequently and in line with the neurodegenerative process, astrocytes and microglia appear in the affected regions. Under physiological conditions, microglia are in a resting state and contribute to innate immune responses by producing anti-inflammatory and neurotrophic factors [41] (Figure 2). In brains of mhtt carriers, microglia are activated even before onset of symptoms [42]. Based on ferritin accumulation and Iba1 immunostaining, an increased microglial activation was also shown in the R6/2 mouse model [43].

In this paradigm, microglial activation correlates with the severity of disease progression and with loss of dopamine D2 receptor binding sites [44, 45]. This could be proven by PET studies using a ligand for the peripheral benzodiazepine receptor (PBR), PK-11195, visualizing activated microglia [46]. The presence of activated microglia induces a cascade of proinflammatory cytokines [47]. Among these are IL-6, IL-12, and tumor necrosis factor alpha (TNF*α*). As downstream effects, an increase of caspase activity, intracellular calcium levels, and the production of reactive oxygen species and nitric oxide could be detected [48]. 

Yet, microglia do not generally exert negative effects. They may release neurotrophic factors that are able to induce neuroprotection and they support clearing cell debris as well as toxic proteins that may worsen degeneration upon accumulation. However, concurrently, microglia with their proinflammatory functions may also damage and remove healthy neurons thus supporting a “bystander” pathogenic process [49] (Figure 2). 

### 3.2. Mutant Huntingtin (mhtt) and Inflammation

To add another level of complexity, mhtt itself is also expressed in microglia [50]. Therefore, in HD, mhtt effects may directly cause inflammation in the CNS and peripheral tissues. Secreted immunomodulatory messengers may then cross the blood brain barrier (BBB) in both directions with inflammation starting in the periphery and spreading into the CNS or vice versa. Studies of Björkqvist et al. confirmed an interaction of both the CNS (“central”) and the peripheral immune system in HD by detecting increased IL-6 and IL-8 levels in plasma and striatum [23]. Since there are no direct alterations at the BBB [51], one might assume that mhtt probably induces ubiquitous changes peripherally as well as in the CNS.

In addition, R6/2 mice show expression of mhtt in other glial cells, mainly in astrocytes, thus reducing the neuroprotective function of these cells. This further underlines the concept of a functional role of inflammation in HD [50, 52].

### 3.3. Macrophages

Macrophages are critical effectors and regulators of inflammation and the innate immune response. In lymphoid and nonlymphoid tissues, their main function consists of phagocytosis and production of growth factor as well as inflammatory cytokines [53]. Macrophage-like cells are part of a diverse group of cell types mediating immune responses. Among these are patrolling monocytes, monocyte/dendritic cells, and CNS infiltrating macrophages. Monocytes circulate and produce inflammatory cytokines. They can differentiate into proinflammatory DCs or macrophages. Classical DCs are antigen-processing and presenting migrating cells that regulate T-cell responses [54, 55].

More recent findings further refined this concept and indicate that macrophages can be differentiated into M1 and M2 subtypes [53, 56]. Activating stimuli of M1 macrophages include IFN-*γ* and IL-4. Consequently, a Th1 response mediated by IL-12 is initiated. In contrast, M2 macrophages rather support Th2 responses in the immune system which are associated with the expression of IL-4, IL-5, and IL-10. These cytokines inhibit macrophage activation but induce antibody production resulting in resolution of inflammation through endocytic clearance and trophic factor synthesis [57]. In general, the concept of M1/M2 macrophages and the Th1/Th2 paradigm postulates that M1 macrophages are assumed to have proinflammatory functions and M2 macrophages may have anti-inflammatory and wound-healing functions. Thus, further studies on M1/M2 macrophages in HD are of great interest. 

Most studies on macrophages in HD and its models investigate migratory capacities. In the BACHD mouse model of HD, an impaired migration of macrophages to an inflammatory stimulus was shown [37]. To demonstrate whether mhtt was necessary for deficits in migration, mice with conditionally deleted mhtt expression in macrophages were generated (BACHD^Tg/+^; CD11b-Cre mice). In these mice, migration of macrophages was not affected which strengthens the hypothesis that migration deficits of macrophages in BACHD mice are governed by mhtt expression in these cells. 

In further studies in humans, blood monocytes from HD patients were isolated to study migration defects. As expected, migration of isolated monocytes and macrophages to chemoattractants was severely impaired [37]. Based on these data, a lack in cytokine and chemokine signaling mechanisms is assumed in HD. This may lead to a chronic increase in proinflammatory cytokines and chemokines in the CNS and consequently microglia activation as well as augmented inflammation.

Comparing the results of isolated primary microglia culture assays, microglial cell lines with overexpression of mhtt, isolated human HD myeloid cells, and* in vivo *studies in HD mouse models, all studies demonstrate and underline the deficits in migration of myeloid cells with mhtt expression. Therefore, the transfer of experiences in HD mouse models into humans seems to be legitimate and helpful in this case. However, despite these detailed studies, the exact nature of mhtt pathogenicity in the immune system, for example, via interacting with the ubiquitin-proteasome system and a capacity for modulating immune and inflammatory responses, is still not completely clarified [58].

### 3.4. The Complement System

One of the most important systems in the innate immune system is the complement system [59], which is often regarded as a connection between the innate and adaptive immune response. Most complement components and receptors are expressed by astrocytes, microglia, and neurons [60, 61]. Thus, the system may be a key factor in several neurodegenerative diseases [59]. In HD, the complement system is activated by peptides such as mhtt. Activation of the complement systems triggers a cascade of processes including cytokine release, enhancing phagocytosis of antigens, attracting macrophages and neutrophils, cell lysis, and, amongst others, production of anaphylatoxins with its central component C3 [62]. In HD, several complement factors, notably C1q, C4, and C3, are expressed in the striatum comprising neurons, astrocytes, and myelin [63]. Yet, it is still unclear which factors initiate this deleterious activation of the complement system. 

### 3.5. Cytokines: IL-6

Increased cytokine production may be caused by dysfunction of microglia and related cell types (monocytes/macrophages). Monocytes of HD patients express mhtt [23]. In turn, mhtt may upregulate NF-kappa B dependent pathways which are crucially involved in IL-6 expression [64]. IL-6 triggers the acute phase response and crosses the BBB [65]. It is primarily produced by monocytes and lymphocytes. On one hand, the function of both of these cells may be directly modified by mhtt. On the other hand, external processes that evoke inflammation, such as tissue damage, may result in IL-6 production. It is even discussed that mhtt itself acts as an antigen and is presented by antigen-presenting cells thus activating inflammation [23].

 IL-6 is upregulated in plasma of HD patients [23, 66]. An excessive IL-6 release was also detected in R6/2 and YAC128 mice. To test the hypothesis that IL-6 may actively influence the disease course in HD, Bouchard et al. administered an IL-6 neutralizing antibody into R6/2 mice. As compared to IgG as control, treatment with the specific antibody diminished weight loss at late stages in R6/2 mice and partially rescued motor deficits on the rotarod (*P* < 0.05) [67].

In further studies, Bouchard and coworkers sought to determine whether cannabinoid receptor 2 (CB_2_) controls IL-6 levels that again contribute to the disease phenotype in the R6/2 model. CB_2_ regulates the production of the proinflammatory cytokines IL-6 and TNF*α* [68–70]. Upon genetical deletion of CB_2_ receptors, which are mainly expressed in peripheral immune cells, IL-6 blood levels were increased [67]. In line, adding the CB_2_ receptor agonist GW405833 significantly reduced plasma levels of IL-6, while levels of TNF*α* were not altered [67]. These studies confirm the hypothesis that IL-6 produced by peripheral immune cells may contribute to pathogenesis in R6/2 mice.

Yet, not only IL-6 but also other inflammatory proteins may be involved in innate immune responses in HD. IL-6 stimulates the expression of another acute phase protein: *α*_2_-macroglobulin (*α*_2_M). *α*_2_M is upregulated in plasma of HD patients, mainly in reactive astrocytes, and therefore influences immune proteins and cytokines. Based on this pathway, *α*_2_M takes part in both the local CNS and peripheral immune system in HD and might contribute to progression of neurodegeneration HD [66, 71].

In concert, these factors may influence astrocytes which are able to amplify inflammatory responses [72]. 

### 3.6. Cytokines: IL-1*β*

IL-1*β* is not only secreted by microglia and macrophages but also typically expressed in dendritic cells (DC) [72, 73] and is verifiably increased in several mouse models of HD and in the serum of HD patients. Notably, brain lysates of R6/2 HD mice display significantly higher levels of IL-1*β* than wild-type control [23, 74–76]. Amongst other cytokines, IL-1*β* may augment inflammatory signals in the CNS. Knockout mice with a deletion of the chemokine receptor type 2 (Ccr2) show impaired migration of monocytes and macrophages. In these mice, serum levels of IL-1*β* and IL-6 were elevated [77, 78], and the mice were unable to induce an effective adaptive immune response [79, 80]. Thus, the reason for elevated IL-1*β* and IL-6 levels in premanifest and manifest HD patients as well as in mouse models may be due to migration deficits in immune cells. These deficits may induce a dysregulated signaling pathway leading to aggregation and elevation of, for example, IL-1*β* levels [37]. In turn, this may directly cause neuronal dysfunction: IL-1*β* itself is able to directly induce neurotoxicity via activation of tyrosine kinases and phosphorylation of NMDA receptors involving the NF-kappa(*κ*)B pathway [81].

Further transferring these results to HD, elevated IL-1*β* levels may additionally lead to deficits in clearing bacterial and viral infections probably further advancing degenerative processes in the CNS and the periphery [37]. 

### 3.7. Genes/Toll-Like Receptors/Inflammasomes

Inflammatory responses are initiated by different receptors, among others including the Toll-like receptors (TLRs). TLR activation generally promotes the expression of proinflammatory cytokines and may induce neuronal damage by changing the environmental conditions in the organisms [82]. TLRs are upregulated in many neurological disorders influencing not only microglia but also astrocytes, oligodendrocytes, and neurons [83]. 

These TLRs recognize pathogens and are highly expressed on macrophages and microglia. TLR activation evokes NF-*κ*B activation resulting in increased transcription of proinflammatory cytokines [84]. Some TLRs, for example, TLR4, which recognizes lipopolysaccharide (LPS), activate microglia [85]. Hereby, DCs, normally not present under healthy conditions, secrete inflammatory mediators such as TNF*α* and IL-1*β* which may affect function of the blood brain barrier thus further contributing to a pathogenic role of DC (see above). The recent evaluation of HD-relevant genes in the HD research crossroads database [86] investigated several new pathways in HD especially pointing at the importance of TLR. There are, for example, 17 genes in TLRs pathways which were analyzed in different studies and which could be merged in the HD crossroads analysis [86].

TLRs are only one of multiple molecular mechanisms associated with the pathogenesis of HD that are up to now only marginally studied. Other factors of interest include proteins involved in cell cycle, RNA splicing, or novel genetic modifiers. Consequently, TLRs may trigger neuroinflammation in a different way. Inflammasomes recruit and activate caspase-1, thereby complementing TLR signaling to generate IL-1*β* and IL-18 and mediating neuroinflammation [84].

Activation of inflammasomes is discussed as central to the pathology of several neurodegenerative as well as autoinflammatory diseases. Inflammasomes are multiprotein complexes which activate immature forms of IL-1*β* and IL-18 into active mature cytokines. This process is caspase-1 dependent [87]. Inflammasomes are activated by the cytoplasmic presence of microbial components, tissue-injury products, or inflammation-associated substances [84]. Its activation is essential in triggering IL-1*β*-driven inflammation and in line also IL-1*β*-driven IL-17 production. In view of previous data on the role of IL-1*β* in HD (see above), inflammasome-derived activation of IL-1*β* may constitute an interesting new target in future research on the role of the immune system in HD. 

In summary, the first line of inflammation in HD undoubtedly involves the innate immune system. Yet, in HD, not only changes in the innate immune system but also altered adaptive immune responses may occur. 

## 4. Role of the Adaptive Immune System in Huntington's Disease

In general, the immune response involves a balanced interaction between the innate and adaptive immune system. Yet, to date, it remains very difficult to exactly determine the initiation point of an adaptive immune response in HD or its animal models.

### 4.1. Dendritic Cells

Dendritic cells (DCs) are antigen-processing and presenting cells. In the immature state, they harbor a high phagocytic activity in contrast to mature cells with a high cytokine producing capacity [54, 55]. DCs have a high migratory capacity and are only short living [88, 89]. Conversely, brain microglia, the macrophage/DC of the CNS, are self-renewing and arise from fetal myeloid progenitors. They prime T-cell responses as a main arm of the adaptive immune system and secrete inflammatory mediators such as TNF*α* and IL-1*β*.

In 1994, a subset of CD11c^−^ immature DCs, with low MHC class II expression were identified. Called plasmacytoid DCs (pDCs), they induce Th2 cell differentiation in response to certain stimuli and are specialized to respond to viral infections with production of type I interferons [90, 91].

Both, myeloid DCs and pDCs may support neuroinflammation. Chronic neuroinflammation includes not only extended activation and proliferation of microglia, as mentioned previously, but also increased levels of proinflammatory cytokines. This prolonged inflammation affects the BBB and impairs its integrity. Normally, the BBB protects the brain from circulating immune cells such as DCs. In HD, the chronic neuroinflammation may weaken the BBB thus leading to infiltration of DCs that may additionally contain mhtt and further foster pathogenic immune responses [23, 92]. 

In summary, the influence of myeloid DCs and pDCs in pathology of HD is critically discussed. There is still a lack of good markers to distinguish between monocytes and bona fide DCs. Thus, a specific effect of DCs in HD cannot be delineated to date. 

### 4.2. T Cells

Elevated T-cell responses and a shift in CD4^+^ and CD8^+^ cell populations were observed in different neurodegenerative disorders. For example, CD4^+^ T cells were found in the substantia nigra in PD patients and there was an alteration of CD4^+^ and CD8^+^ T cells in AD [93, 94].

In contrast, T cells were not numerically increased in postmortem human HD tissue [38]. Silvestroni and coworkers analyzed the striatum, cortex, and cerebellum from postmortem HD patients using quantitative real-time PCR. As a result, they assumed that neuroinflammation in HD is solely based on interaction of microglia, neurons, and macroglia. In contrast, some evidence for a role of T cells in HD has emerged from studies that measured elevated T-cell responses to specific antigens as well as shifts in CD4^+^ and CD8^+^ cell populations in the CNS [93]. However, functional studies and comprehensive immunological analysis on T cells in HD have not been largely performed to date. In summary, the involvement of T cells as a causative factor in disease pathology is still difficult to verify, and further studies are needed to decipher the exact role of different T-cell subsets in HD, especially with a focus on regulatory T cells (see also what follows for Th2 cells).

### 4.3. IL-4

IL-4 is an anti-inflammatory cytokine, which is involved in the adaptive immune system and is regarded as a signature factor of “Th2” T helper cells. Moreover, IL-4 induces B-cell class switching, upregulates MHC II class production and is also involved in tolerance and induction of regulatory T cells. Consequently, IL-4 decreases the production of Th1 cells, macrophages, IFN-*γ*, and IL-12. In line, IL-4 increases the production of M2 macrophages under conditions of IL-10 and TGF-*β* secretion while inhibiting classical activation of macrophages into M1 cells [95]. In concert with the repair function of M2 macrophages, IL-4 actions result in diminished neuroinflammation. Thus, IL-4 may be an interesting regulatory factor in HD.

Yet, the exact role of regulatory cytokines T cells and Th2 cells in neurodegenerative diseases is not exactly known. There may be beneficial effects on regeneration involving BDNF or IL-4, but detailed mechanisms are still not completely understood [96]. In plasma samples from HD mutation carriers, IL-4 levels were significantly increased compared to control subjects [23]. This fact emphasizes the hypothesis that the adaptive immune system does play a role in HD. The increase of IL-4 and in line IL-10 both in HD patients and in the CNS as well as peripherally also in HD mouse models was mainly seen in late stages of the disease [23]. This may implicate an adaptive response to chronic immune activation, possibly involving monocytes/macrophages and “Th2” cells. 

In summary, the data mentioned previously support meaningful interactions between immune cells and neurodegeneration in HD. Of note, migration deficits seen in immune cells in both, HD patients and mouse models, may be an initial step in early immunological changes and may lead to elevation of proinflammatory cytokines and chemokines in HD. 

## 5. Conclusions

In summary, there is a large body of evidence for a pivotal role of neuroinflammation in the development of several neurodegenerative diseases. Yet, the exact underlying inflammatory pathomechanisms and the definite impact of the innate and adaptive immune system in HD pathology are still not fully understood. It is not verified yet if changes in the immune system which may trigger or may be triggered by, for example, neuronal death are the cause or the consequence of HD pathology. However, an activation of the immune system in HD is not doubted and was clearly proven by the elevated expression of cytokines such as IL-6 in mouse models and symptomatic as well as presymptomatic patients. Furthermore, activation of CNS innate immune cells in HD, such as microglia and astrocytes, is one of the universal components of neuroinflammation, and these cell types are directly implicated in the pathogenesis of several neurodegenerative diseases. Therefore, the contribution of inflammation to neurodegeneration in HD is strongly suggested but not definitely demonstrated. 

## 6. Outlook

In view of an aging community and a growing incidence of neurodegenerative diseases, the need for effective therapies rapidly increases. Changes in microglial properties are not yet well understood and thus remain in the center of ongoing investigations. Still these cells constitute an interesting new therapeutic target in HD.

A first idea to reduce neuronal damage via interfering with the immune system was the use of minocycline. As a caspase inhibitor, minocycline aims at the reduction of TNF*α*, IL-1*β*, and inducible nitric oxide synthase (iNOS). In the R6/2 mouse model of HD, the compound led to a significant delay in disease progression. A small study in HD patients has also shown positive effects with this well-tolerated, and safe drug [97].

Assuming inflammatory mechanisms as a key player in HD pathogenesis, anti-inflammatory treatments with Cox2 inhibitors were analyzed. Yet, there was no neuroprotective effect in the R6/2 and N171-82Q transgenic mouse models of HD although in theory folding of mhtt should be improved, inflammatory pathways should be less activated and pro-apoptotic signaling mediated by NF-*κ*B should be reduced [98].

Administering a compound that inhibits the proinflammatory cytokine TNF*α* may also reduce neuroinflammation hereby delaying disease progression in HD. TNF*α* inhibitors, for example, etanercept, are biologic drugs. However, etanercept is a large molecule and therefore does not cross the BBB [99]. This may be the reason why this approach displayed no convincing therapeutic effect in mouse models of neurodegenerative diseases [100].

Another hypothesis-driven anticytokine approach may be the treatment of HD with antibodies against the IL-6 receptor (IL-6R, tocilizumab). IL-6R antibodies are a widely used therapy for rheumatoid arthritis. While this disease is not directly comparable to HD, it shows that this approach is very well tolerated, and first data on longer-term treatment in CNS disease-like neuromyelitis optica were recently published [101]. In view of the pivotal role of IL-6 in HD, further studies with treatment options targeting IL-6 mediated pathways are clearly warranted. 

Finally, an auspicious approach in the therapy of HD may be the application of the fumaric acid ester dimethylfumarate (DMF) as a modern immunomodulator with pronounced effects on macrophages [102]. The compound was recently approved for the treatment of relapsing remitting multiple sclerosis in the USA. Own studies further revealed that DMF potentially exerts neuroprotective effects via induction of the transcription factor “nuclear factor E2-related factor 2” (Nrf2) and detoxification pathways [103]. Since oxidative stress and free radicals are, amongst macrophages, known to support neurodegeneration, we thus investigated the therapeutic efficacy of DMF in R6/2 and YAC128 HD transgenic mice [104]. After DMF treatment, there were significant differences in survival, motor impairment, weight loss, and parameters of neuronal degeneration in favor of DMF therapy. Initiating a phase II study with DMF in HD patients would be a promising next step in treating HD probably targeting both, immune cells and detoxification pathways.

In summary, there is no doubt that the importance of the immune system in neurodegenerative diseases is increasingly recognized. The near future will certainly see further studies targeting immune responses in HD as a therapeutic approach.
